# Recent Advances in Meningioma Immunogenetics

**DOI:** 10.3389/fonc.2019.01472

**Published:** 2020-01-08

**Authors:** May Al-Rashed, Kara Foshay, Malak Abedalthagafi

**Affiliations:** ^1^Department of Clinical Laboratory Sciences, College of Applied Medical Sciences, King Saud University, Riyadh, Saudi Arabia; ^2^Inova Neuroscience and Spine Institute, Inova Health Systems, Falls Church, VA, United States; ^3^Virginia Commonwealth University School of Medicine, Inova Campus, Richmond, VA, United States; ^4^Genomics Research Department, Saudi Human Genome Project, King Fahad Medical City, King Abdulaziz City for Science and Technology, Riyadh, Saudi Arabia

**Keywords:** meningioma, NGS, genomics, immunotherapy, neuro-oncology, personalized medicine

## Abstract

Meningiomas are relatively common, and typically benign intracranial tumors, which in many cases can be cured by surgical resection. However, less prevalent, high grade meningiomas, grow quickly, and recur frequently despite treatment, leading to poor patient outcomes. Across tumor grades, subjective guidelines for histological analysis can preclude accurate diagnosis, and an insufficient understanding of recurrence risk can cloud the choice of optimal treatment. Improved diagnostic and prognostic markers capable of discerning between the 15 heterogeneous WHO recognized meningioma subtypes are necessary to improve disease management and identify new targeted drug treatments. In this review, we show the advances in molecular profiling and immunophenotyping of meningiomas, which may lead to the development of new personalized therapeutic strategies.

## Introduction

The dura mater, arachnoid mater, and pia mater, are the three protective membranes that surround the brain and spinal cord, together forming the meninges (1–3). Tumors arising from this tissue, called meningiomas, are the most common primary intracranial tumors of the central nervous system (4). Studies in genetically engineered mice suggest that meningiomas arise from prostaglandin D2 synthase (PGDS) expressing arachnoid cells which protrude through the dura on arachnoid villi (5). Resulting tumors are most commonly found in intracranial, intraspinal, or orbital locations, with intraventricular and epidural tumors presenting less often. Rarely, extradural meningiomas can also occur (4). Though a definitive cause has yet to be determined, exposure to radiation and inherited Neurofibromatosis syndrome can predispose affected individuals to meningioma. In the United States, meningioma is diagnosed at a rate of ~98 per 100,000 persons, at a median age of 55 years (1). A hormonal influence on tumor formation and progression is suggested by the high prevalence of low grade meningioma in women compared to men (3:1 in the brain; 6:1 in the spine), decreased incidence before puberty and after menopause and rapid tumor growth during pregnancy (6).

Diverse in nature, the World Health Organization currently recognizes three grades of meningioma further divided into 15 subtypes (4). Approximately 80% of these are benign, WHO grade I tumors, for which surgical resection is often curative and 10-years overall survival is estimated at 80–90% (7). However, Grade II and III meningiomas, which represent 15–18 and 2–4% of all meningiomas, respectively, are difficult to treat due to aggressive growth and frequent recurrence, often within 5 years. Indeed, Grade III malignant meningiomas harbor a poor prognosis, with 10-years overall survival averaging 14–34% (7). Interestingly, in contrast to low grade meningioma, these high-grade tumors are more common in men than women.

Among the nine subtypes of Grade I meningioma, the most common include meningothelial, fibroblastic, and transitional (a combination of the previous two) (4). Grade II meningiomas are diagnosed based upon a mitotic count of 4–19 per 10 high powered fields with the presence of brain invasion, or by the presence of at least three morphological criteria; high cellularity, small cells with a high nuclear to cytoplasmic ratio, sheeting, necrosis, or prominent nucleoli. Within these criteria, tumors are classified as clear cell, chordoid, or atypical meningioma. Tumors with more than 20 mitotic events per 10 high powered fields, brain invasion, necrosis, and loss of typical architecture are diagnosed as either anaplastic, rhabdoid, or papillary Grade III meningioma.

**Figure 1:**
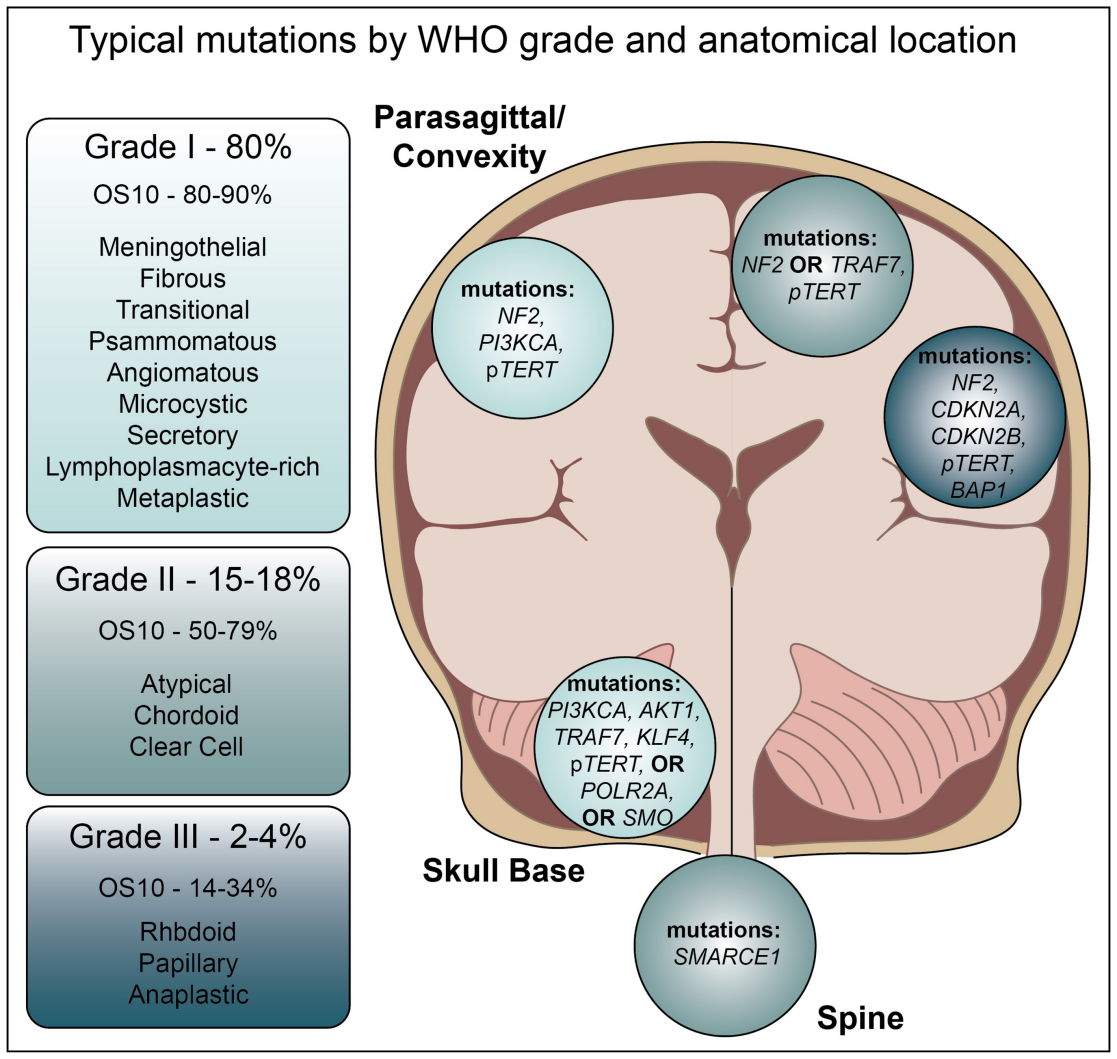
Typical mutations by WHO classification and anatomical location.

Despite WHO grading criteria, meningioma diagnosis can be complicated. Angiomatous meningiomas represent 2% of Grade I tumors and are generally easily characterized by a predominance of blood vessels. However, this particular subtype, while benign, can present with histological evidence of nuclear atypia and microcystic features, leading to unnecessary concern for progression (8). Fibrous meningiomas may be confused with schwannoma or solitary fibrous tumors (hemangiopericytoma), while Grade I microcystic or Grade II clear cell tumors may resemble hemangioblastoma. Grade III anaplastic meningioma may resemble sarcoma or carcinoma, and uncommon presentations, such as extradural tumors, may influence the differential diagnoses. Given the subjectivity of the histological criteria, grading may require additional observers or the use of anatomical location to aide in diagnosis. Grade II and grade III meningiomas are most often intracranial, clear cell meningiomas are commonly intraspinal, and anterior cranial base and intraventricular lesions exhibit decreased progression free survival (9). In addition, these locations are associated with distinct genomic profiles that influence the aggressive nature of the tumor and chance of recurrence (Figure 1).

Standard of care treatment for meningioma is based primarily on patient status and WHO grading when biopsy is available (3). As there are no screening criteria for meningioma, findings in asymptomatic patients are often incidental (3). For these patients, observation may be the primary treatment approach. Surgery is the first line of therapy for symptomatic meningiomas, and extent of resection is the most significant factor in determining the chance of recurrence. For Grade II patients undergoing gross total resection, the clinical benefit of fractionated radiation therapy vs. observation is still unclear (Clinical Trial NCT03180268). However, when gross total resection is not possible (independent of tumor grade), when surgery is contraindicated, either SRS of FRT are used to improve outcomes. Though limited in efficacy, systemic adjuvant chemotherapy may also be used to treat aggressive high grade tumors. Unfortunately, across tumor grades, the chance of recurrence can vary widely, and current algorithms for predicting disease progression cannot discern which patients are at highest risk. However, improved methods utilizing cytogenetics, mutational profiles, and epigenetics may improve our capacity for effective, patient-specific, treatment of meningioma.

## Cyotgenetics

Many meningiomas possess a normal karyotype, with an overall low incidence of genomic alterations (i.e., somatic copy number alterations (SCNA), rearrangements, mutational burden) (10–13). However, these disruptions increase in accordance with tumor grade and aggressiveness. More than half of all identified genomic alterations involve the neurofibromin gene (*NF2*), which is known to underlie inherited Neurofibromatosis syndrome. Indeed, the most significant, and often the only, SCNA in meningioma is chromosome 22 monosomy, which is present in ~56% of cases and leads to loss of the genomic locus containing *NF2* (22q12.2) (11, 12, 14). Among benign meningiomas, those carrying *NF2* alterations are more likely to progress than those with a normal karyotype. In addition, the frequency of *NF2* aberrations increases with tumor grade.

Loss of heterozygosity on chromosome 1p is the second most frequent cytogenetic abnormality seen in meningioma (~16%) (15). Characterization of the smallest region of overlapping deletion on this chromosome, which spans ~3.7 megabases, identified 59 genes, 17 of which have putative tumor suppressive functions based on gene ontology. The protein methyltransferase and tumor suppressor, *RIZ1*, is located on chromosome 1p, and studies implicate its loss of expression in meningioma progression (6). Loss of the *CDKN2A/CDNK2B* locus on chromosome 9q is also a relatively common event during progression from grade II to III (16). Interestingly, recent efforts also identified a recurrent amplification of this locus, within grade I tumors (17). These data suggest that levels of p16 and p15, the proteins encoded by *CDKN2A* and *CDKN2B*, may hold prognostic significance and/or represent a promising therapeutic target.

Loss of chromosomes 6q, 9p, 10q, 14, and 18q, as well as gains in 17q and 20q are also observed, though with lower frequency (11, 13, 16). In a multivariate analysis of 302 meningiomas, alterations within the chromosomal set of 1p, 1q, 7, 9, 10, 14, 18, and 22 were associated with significantly higher incidence of relapse (9). This study went on to stratify cases based on the number of cytogenetic abnormalities, demonstrating that tumors with a “complex karyotype,” consisting of two or more affected chromosomes, were associated with significantly decreased recurrence free survival (RFS) compared to those with a single affected chromosome or a normal diploid karyotype (9). Another similar, but distinct study of chromosomal characterization demonstrates that the presence of chromosome 5 polysomy, even in the presence of multiple polysomies on chromosomes 20, 6, 12, and 13, can distinguish angiomatous meningioma from more aggressive forms of the disease (18). Interestingly, this cytogenetic profiles seems to be independent of *NF2* mutation, as well as other common driver mutations identified in grade I meningioma.

Several other individual amplifications in genes including, *FGF3, ZNF217, ZNF331, CDK4, ERBB3, LRG5, MDM2, NACA, PTPN11, WIF1, PDCD1, TLX1, ARFRP1, GNAS, SS18L1, FoxA1, FGF6*, and *FGF10*, have also been identified, though evolutionary analysis of these tumors reveals that SCNA likely precedes such driver mutations, leading to both inter- and intra-patient heterogeneity in mutational profiles (17). Interestingly, the identification of spatial heterogeneity among multiple tumors from the same patients, suggests that distinct microenvironments may influence tumor formation and growth (11). However, for clinical risk analysis, the overall SCNA burden of any given tumor remains one of the strongest predictors of RFS.

## Genomics NGS

### *NF2*-Mutated Meningioma

As high grade meningiomas are associated with rapid disease progression and poor prognosis as compared to low grade, recent efforts in next-generation sequencing have sought to identify prognostic biomarker to differentiate between tumors of varying grades, and those that may correlate with treatment response. With a low mutation rate (~3.5 mutations per megabase) compared to other cancers (17), these efforts highlight the challenges in managing these heterogeneous tumors. Similar to cytogenetic analysis, these studies identified *NF2* mutations as the predominant alteration in both spontaneous (~60%) and Neurofibromatosis syndrome associated (~40%) of tumors (16), at a frequency of 43% in low grade, and nearly 80% in high grade tumors (11). Interestingly, *NF2* mutations were more common in the cerebral convexities and posterior skull base tumors than those found in other anatomic locations (19). While no other co-mutations were identified in more than 13% of cases, single mutations in *CREBBP, PIK3CA* (R108H), *PIK3R1, BRCA1*, and *SMARCB1* were also observed (19). Unfortunately, within *NF2* mutated meningiomas none of these identified mutations can predict the chance of recurrence, which can vary widely.

More recently, *TERT* promoter mutations have been reported in ~6% of all meningiomas, with ~80% of these also harboring alterations (mutations or deletions) at the *NF2* locus (20). Similar to the overall mutational burden, *TERT* mutations increase with tumor grade. In grade I meningioma, *TERT* C228T and C250T mutations are linked with transformation to higher grades (20), prompting many scientists and clinicians to consider standardized testing for these specific changes. Further studies demonstrate that the presence of C228T and C250T correlates with increased *TERT* mRNA and functional increases in telomerase activity (21), and in Grade II or III tumors, univariate analysis revealed a significant association with decreased progression-free survival (PFS, median 12.5 vs. 26 months, *p* = 0.004) and overall survival (OS median 26 vs. 46 months, *p* = 0.009) (22). *In vitro, TERT* mutated meningioma cells show decreased TERT activity in response to YK-4-279, a small molecule inhibitor of ETS transcription factor, suggesting a novel potential strategy for targeting these aggressive tumors. In addition to the C228T and C250T mutations, recent efforts using targeted sequencing approaches identified an additional *TERT* promoter in the known hotspot G124A, which like other *TERT* mutations seems to correlate with poor prognosis (23).

### Non-*NF2* Meningioma

Non-*NF2* mutated tumors, which are predominantly benign, chromosomally stable, and often located in the anterior, medial, or skull base regions, possess a distinct mutational landscape (Figure 1) (19). Recent high throughput sequencing efforts suggest an average of only 1.56 ± 1.07 genomic alterations (GAs) per patient (23). The pro-apoptotic E3 ubiquitin ligase, tumor necrosis factor receptor-associated factor 7 (TRAF7) is mutated ~24% of all meningiomas (19, 24). Such mutations typically occur in the C-terminal WD40 protein interaction domain, suggesting they may alter protein-protein interactions with MAPK and NF-kB family members (25). While *TRAF7* mutation is mutually exclusive with *NF2* mutations, it nearly always occurs with the PI3K activating E17K mutation in *AKT1*, or in *KLF4* (K409Q) (19, 24).

The E17K mutation in *AKT1* leads to constitutive activation of its gene product, protein kinase B, and stimulates downstream mTOR signaling (12, 19, 26). Known to be oncogenic in many other cancer types (27), this mutation is found in 7–12% of grade I meningiomas (3, 11, 12, 19), is enriched in the meningothelial subtype (11), and is predictive of decreased progression free survival in olfactory groove tumors (28). Altering the same signaling pathway *PIK3CA* mutations are also found in ~7% of non-*NF2* tumors, and are mutually exclusive with *AKT1* mutation (26). Recent targeted sequencing of this gene revealed three novel non-synonymous mutations, A3140T and A3140G which are reported as pathogenic, and C112T, which is also predicted to be pathogenic (23). Indeed, increased PI3K signaling at the protein level is associated with aggressive behavior, especially within malignant meningioma (29), suggesting that therapeutics targeted toward this pathway may be beneficial.

Targeted sequencing of cancer genes in a cohort of 71 meningiomas recently identified two novel missense mutations in *FGFR3*, T932C, and G1376C, both of which were predicted to be pathogenic (23). The identification of these mutations in patients with skull base WHO grade I tumors receiving no adjuvant therapy and no recurrence, suggests that *FGFR3* mutation may be indicative of improved prognosis. This hypothesis warrants further investigation in larger patient datasets.

Best known for its role in pluripotency, Klf4 is thought to act as a tumor suppressor in meningioma, being robustly expressed in low grade tumors and downregulated in anaplastic tumors (30). At the genetic level, *KLF4* is mutated in ~12% of grade I meningiomas (3, 11), virtually all of which are of the secretory sub-type and also harbor *TRAF7* mutations (31). All identified *KLF4* mutations result in a K409Q substitution within the DNA binding domain, which likely alters or blocks key protein functions (32).

Mutations in the gene smoothened (*SMO*), which result in L412F or W535L substitutions lead to functional activation of Hedgehog signaling in meningioma (3, 11, 12, 19). These mutations are present in ~5.5% of grade I meningiomas, and are mutually exclusive with *TRAF7, KLF4*, and *AKT1* mutations (3, 26). Interestingly, meningiomas with the L412F mutation are more likely to recur (3), and are enriched at the midline, perhaps reflective of the key role Hedgehog signaling plays in hemisphere separation during development (19). Mutations in the Hedgehog family member *SUFU* are also found at low frequencies in sporadic meningiomas, though germline mutations are also present in familial meningioma (33). Additional hedgehog family germline mutations occur in *SMARCE1* and *SMARCB1*, though these carry less risk of recurrence than familial *NF2* mutations (34–36).

Exclusive of *TRAF7, AKT1, PIK3CA, KFL4*, and *SMO*, mutations in *POLR2A*, which encodes DNA-directed RNA polymerase II subunit RPB1, are found in 6% of meningiomas (33). Inactivating somatic and germline mutations, or gene deletions in the *BAP1* tumor suppressor gene are found specifically within high-grade rhabdoid meningioma (37). In addition, loss of BAP1 correlates with tumor aggressiveness and decreased time to progression. Though previously not identified, a recent study found ARID1A mutations in nearly 12% of a 50-patient cohort (38). Deleterious mutations in this gene, as well as the other SWI/SNF components SMARCB1, SMARCA4, and PBRM1, were found in 16% of all anaplastic meningiomas (38).

## Methylation

The current WHO classification system for meningioma is relatively subjective and alone often fails to accurately predict disease progression. Interestingly, some mutations may drive epigenetic changes, such as inactivation of the SWI/SNF complex, which disrupts its balance PCR2, leading to altered methylation profiles (38). Further data support the implication of gene methylation patterns as driver of disease formation or biomarkers of progression. Loss of retinoblastoma protein-interacting zinc-finger gene (*RIZ*), which maps to chromosome 1p36, is associated with progression of meningioma (6). This gene produces two proteins, RIZ1 and RIZ2. RIZ1 is a histone 3 lysine 9 methylase, an important regulator of transcriptional repression, and a known tumor suppressor. In pituitary adenomas, methylation of the *RIZ1* promoter region is associated with altered epigenetic profiles and decreased progression free survival (39). Although its role in meningioma is less clear, RIZ1 is expressed in 87.5% of grade I, 38.9% of grade II, and 23.8% of grade III tumors (40). This inverse correlation suggests that RIZ1 methylation, expression, and/or resulting epigenomic profiles may be useful for predicting disease prognosis.

Methylation of other genes is also implicated in meningioma formation. Specifically, hypermethylation of *WNK2* is present in 83% of grade II and 71% of grade III tumors and is associated with loss of gene expression (41). As a negative regulator of cell proliferation, loss of WNK2 is likely associated with more aggressive tumor growth. In another study, researchers designed a highly specific and sensitive system, which independent of WHO grade, could predict meningioma recurrence based on the extent of methylation present in a set of 5 homeobox genes (*HOXA6, HOXA9, PENK, UPK3A*, and *IGF2bP1*) (42). These studies suggest that for tumors such as meningioma, with a low burden of genomic aberrations, epigenetic approaches to classification and biomarker identification may be more fruitful.

Furthering the epigenetic characterization of meningioma, another recent study found that the unique DNA methylation profiles between intracranial tumors and within subtypes of meningiomas represents an additional means for tumor classification. Unsupervised clustering of DNA methylation data is capable of definitively segregating all meningiomas, across grades, from other skull tumors (43). Further delineation of these profiles identified two major epigenetic patterns, groups A and B, which are further divided into 4 and 2 “methylation classes (MC),” respectively. Methylation group A contains three benign MCs designated as ben-1, ben-2, ben-3, and one intermediate MC, int-A. Methylation group B is comprised of int-B and one malignant MC, referred to as mal (43). These groups associated with common mutational profiles, with the majority of *NF2* mutated samples clustering in group A and most *Tert* mutations in group B. In addition, these classifications could predict length of progression-free survival (PFS) with higher accuracy that WHO grade alone.

Another recent investigation used regression modeling to generate a methylation profile-based algorithm that could accurately predict 5 year PFS rates (44). Detailed characterization revealed hypermethylation of CPG sites in homeobox or T-box genes in recurrence-prone tumors, although this was not associated with expression level changes. This group went on to generate a meningioma recurrence score, based on the methylome-predictor, extent of resection, and WHO grade that can be used clinically to assess recurrence risk across patients as well as to inform choices for follow-up scheduling and administration of adjuvant therapy (44).

## Expression/Immunoprofiling

While some prognostic protein-level changes are the result of genetic alterations, others represent epigenetic changes or post-translational modifications. Many such proteins are used to aid in diagnosis and grading of meningioma through standard immunohistochemical (IHC) approaches. However, as higher grade meningiomas necessitate adjuvant therapies, there is also great interest in identification of new protein biomarkers that are predictive of, or correlate with, treatment response.

Standard IHC markers used to distinguish meningioma from other intracranial tumors include progesterone receptor (PR) and epithelial membrane antigen (EMA) (4, 45). However, studies suggest that PR specificity is greatly reduced in high grade meningiomas of the anaplastic, atypical, clear cell, fibrous, and microcystic subtypes compared to grade I tumors (20 vs. 85%). Likewise, EMA expression correctly identifies ~90% of grade I meningiomas, but only 75% of grade III, with even lower rates of specificity for secretory and microcystic subtypes. Because the sensitivity and specificity of these markers is sub-optimal, absence of additional markers, including S100, CD34, MelanA are used to discern meningioma from differential diagnosis of schwannoma, solitary fibrous tumor/hemangiopericytoma (SFT/HPC), or metastatic melanoma, respectively.

Recent studies demonstrate that other markers and combinations thereof may increase the specificity and sensitivity of meningioma diagnosis and grading. Expression of somatostatin receptor 2A (SSTR2A) in combination with EMA is associated with 100% sensitivity and 94.8% specificity for meningioma, regardless of grade or subtype (45). These data suggest that SSTR2A may be a better choice than PR for standard IHC. However, this combination of markers does overlap with synovial sarcoma. Likewise, recent work suggests that the absence of Sox10 (45, 46) and STAT6 (45, 47) are superior approaches to distinguishing meningioma from schwannoma and SFT/HPC.

Other classical markers of tumor growth, proliferation, and angiogenesis have also been examined in meningioma. Expression of VEGF and Ki67 were both found to associated with tumor grade, while COX-2 expression correlated with the extent of brain invasion (48). MMP-9, a matrix metalloproteinase, expression was also found to correlate with brain invasion (49). It is enriched in high grade meningiomas, and found to associate with high levels of peritumoral brain edema (50). Tyrosine kinase signaling is often associated with tumor progression. In the majority of meningiomas, both EGFR and its ligand EGF are overexpressed (51, 52). However, activation of this pathway, as evidenced by EGFR phosphorylation is enriched within higher grade tumor samples (51, 52). PDGFRB and its ligand PDGFBB are also overexpressed in meningioma, with higher levels observed in high grade atypical tumors as compared to benign (53).

Analysis of immune cell infiltration within a tumor can also aide in diagnosis, though this approach also holds potential for identification of biomarkers associated with progression, treatment response, or sensitivity to targeted therapeutics. Most low grade meningiomas possess a high percentage of CD-3^+^ T-lymphocytes but relatively few CD20^+^ B cells, however, across tumor grades, these populations are greatly enriched compared to those seen in peripheral blood mononuclear cells (PBMC) (54, 55). Flow cytometry analysis reveals evidence of class switching in B cells, as well as an increased percentage of CD8^+^ cells compared to CD4^+^ T cells, and a prevalence of CD45RO^+^/CD45RA^−^ effector cells compared to naive T cells (54). Combined these data suggest that most infiltrating immune cells are antigen experienced. Further identification of T cells expressing the checkpoint inhibitors PD-1 and TIM-3, are suggestive of T cell exhaustion. Within anaplastic meningioma, a decrease in both CD4^+^, CD8^+^, and PD-1^+^ T cells, is observed with a concomitant increase in FoxP3^+^ T-regulatory cells (TRegs) (55). This immune cell phenotype, also observed in other tumor types, is associated with tumor-mediated evasion of the immune system (56).

Within tumor cells, expression of PD-L1, the PD-1 receptor ligand, increases with WHO tumor grade (17, 55, 57, 58). However, overall mRNA or protein levels, as analyzed by RT-PCR, ISH, IHC, and flow cytometry varies widely from study to study. Du and colleagues report high levels of PD-L1 mRNA which correlated to protein expression levels, in ~40% of grade I, 60% of grade II, and 77–88% of grade III meningiomas (55), while Everson and colleagues only identified PD-L1 in 25% of grade III cases, with no IHC expression detected in grade I or II cases (17). One potential source of these observed differences in PD-L1 expression may arise from the specific populations of cells analyzed. In particular, there is evidence that in addition to tumor cells, tumor infiltrating macrophages may comprise upwards of 50% of the PD-L1 expressing cells, a ratio which varies extensively from patient to patient (57).

Given that PD-L1 expressing cells vary in phenotype from tumor to tumor, it is unsurprising that the literature presents conflicting reports on the link between PD-L1 expression and mortality. While Du reported that PD-L1 was not an independent predictor of outcome, Han and colleagues used univariate analysis to identify the proportion of PD-L1 tumor cells as a significant predictor of outcome (57). Importantly, this group used the macrophage marker, CD68, to exclude tumor infiltrating cells. As this receptor ligand is the target of several new drugs, further investigation into the predictive link between PD-L1 expression and therapeutic sensitivity is important. Indeed, based on evidence of their expression, other checkpoint inhibitors, such as those targeting TIM-3 or LAG-3 may be useful in treating (54, 59).

Additional protein biomarkers that represent actionable drug targets in meningioma include EGFR, which is expressed at elevated levels in 93% of analyzed samples (17). Increased TOP2A expression, observed in ~35% of samples, correlates with increasing tumor grade, and is predictive of anthracycline responsiveness (17). Likewise, TOP1 over-expression is observed in 29% of meningiomas and correlates with sensitivity to irinotecan and topotecan, while elevated levels of PDGFR and c-MET are observed in more than 20% of cases (17). Further investigation of patient samples reveals an average of 10 clonal HLA neoantigen mutations per tumors. These tumor-specific antigens are distinguishable from normal tissue and represent a path toward personalized anti-meningioma therapy (11).

## Medical Management

### Traditional Approaches

For many meningiomas maximal safe surgical resection, with or without adjuvant radiation, can be curative. However, for aggressive high grade meningiomas, which often recur at high rates, there are no standard effective medical treatments. In general, conventional chemotherapy had limited efficacy in managing meningioma (60). Hydroxyurea has long been used as an adjuvant therapy for incompletely resected or recurrent meningioma (61, 62). Although the benefits vary widely across patients, studies suggest hydroxyurea may have outcomes equivalent to those using radiation therapy (63). The alkylating agent, temozolomide, which is used as standard of care in medical management of glioma, failed to extend progression free survival in clinical trials of recurrent meningioma (64).

The well recognized effects of hormonal dysregulation on meningioma development and progression led to numerous trials of hormone targeting agents. While the commonly used anti-estrogen therapy, tamoxifen, failed to improve outcomes (60), mixed success has been observed in small trials of the anti-progesterone mifepristone (65–68), suggesting that patient stratification by progesterone receptor expression within the context of a trial may clarify the utility of this approach (60).

Immunohistochemical analysis has demonstrated that canonical cancer-associated tyrosine kinase pathways function at elevated levels in meningioma, driven by overexpression of receptors and/or their cognate ligands. This finding led to trials of tyrosine kinase inhibitors, often used successfully to treat other cancers. In 25 recurrent meningioma patients treated with either erlotinib or gefitinib, both EGFR inhibitors, eight patients showed stable disease (69). However, the remaining patients progressed, with no significant differences in PFS or OS observed. Likewise, a phase II trial of the PDGFR inhibitor, Imatinib, did not identify any statistically significant changes in PFS or OS (70). Unlike these specifically targeted therapies, Sunitinib is a tyrosine kinase inhibitor with activity against PDGFR, as well as, VEGFR and c-KIT. In a phase II trial of 36 recurrent meningioma patients with significant history of adjuvant therapy, sunitinib treatment resulted in increased PFS (median = 5.2 months), and patient response correlated with VEGFR2 expression (71). These data merit further exploration of Sunitinib or pan-TKIs as a treatment for recurrent meningioma.

Despite the encouraging results from sunitinib trial in improving hearing and tumor shrinkage in some *NF2* patients with progressive vestibular schwannomas, treatment with bevacizumab, a drug specifically targeting VEGFR, did not result in clinically significant response in meningiomas. In a retrospective study of 48 meningiomas within 15 neurofibromatosis 2 (*NF2*) patients treated with bevacizumab for vestibular schwannoma, radiographic response to treatment (>20% reduction in tumor volume) was observed in 29% of tumors (72). As many patients had multiple tumors, this corresponded to 1 out of 15 patients. However, this response was short-lived, as only 5 of the 14 meningiomas maintained responsiveness through the last follow-up. A more recent phase II trial of recurrent radiation refractory meningioma demonstrated stable disease as the best response to bevacizumab, with no evidence of radiographic response (73).

Recent identification of somatostatin receptor (SST2) as a highly sensitive marker of meningioma, highlights the potential of the somatostatin analog, octreotide, a candidate therapy. One trial in grade I skull base meningiomas showed long-term stability (mean 102 months) in 5 of 6 patients (74). However, in a phase II study of 12 recurrent or progressive meningioma cases treated with octreotide, only two patients showed long term PFS (75). Likewise, in a trial of nine high grade meningioma patients treated with octreotide (76), and in a larger trial of an alternative somatostatin analog, pasireotide (77), radiographic response was not observed in any patients, and no significant increase in progression free survival was detected. Indeed, a recent *in vitro* analysis of 81 patient-derived meningiom cell lines revealed significant anti-proliferative effects octreotide, but no apoptotic response (78). These data support the clinical observation of stable disease with the absence of tumor shrinkage. Although inhibition of SST2 failed to improve clinical outcomes, expression levels of SST3 have been shown to correlate with increased overall survival, perhaps meriting further investigation (77).

### New Approaches and Clinical Trials

**Figure 2:**
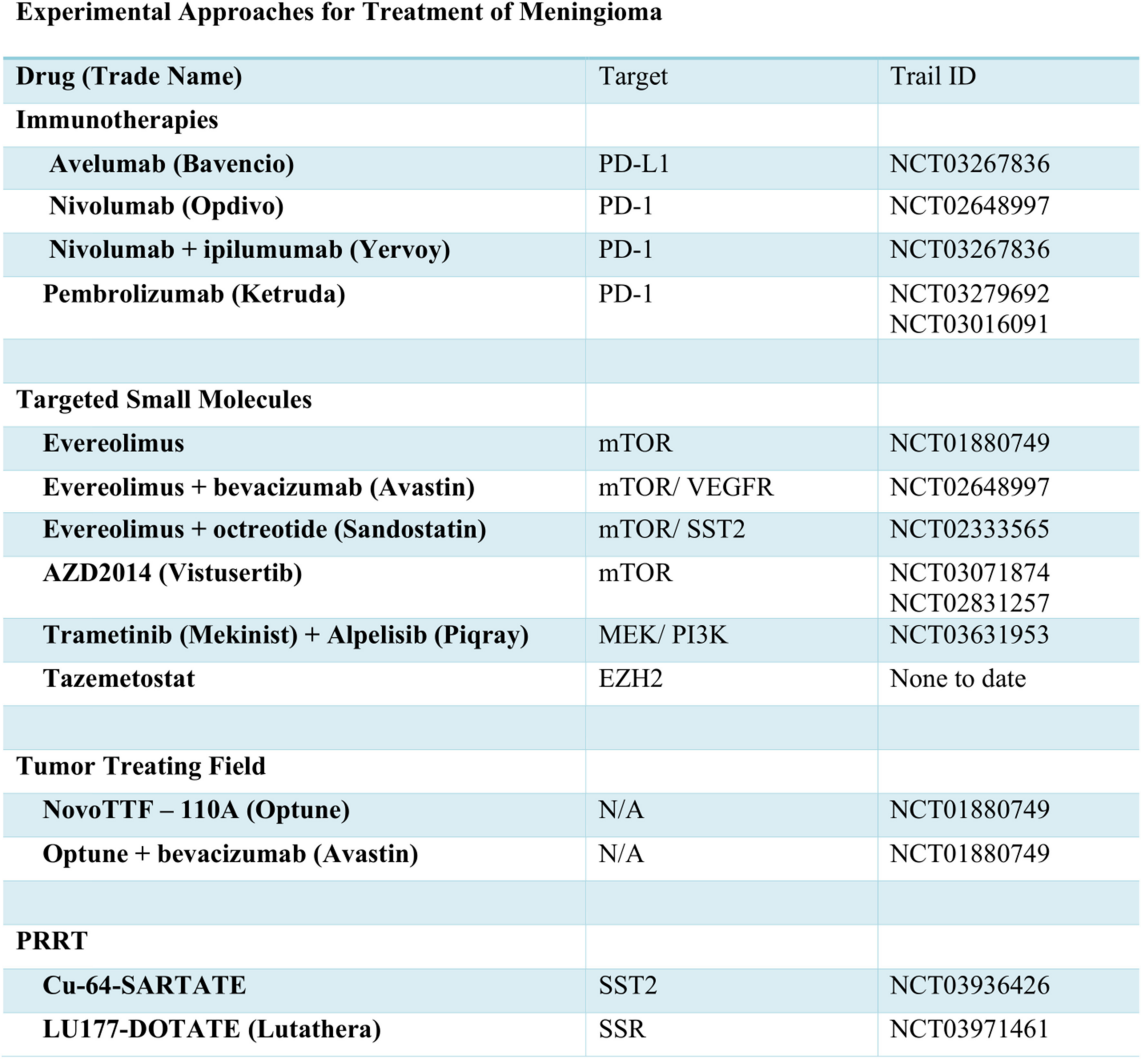
Experimental approaches for treatment of meningioma.

Traditional approaches have used cytotoxic chemotherapy for treatment of meningioma. Currently, the alkylating agent, Trabectedin, is under investigation for efficacy (Figure 2). Aside from transcriptional inhibition, the mechanism of Trabectedin is not completely understood, though *in vitro* studies demonstrate decreased cell proliferative and massive induction of apoptotic cell death (79). However, in the recently concluded EORTC-1320-BTG randomized phase II clinical trial (NCT02234050), trabectedin failed to improve PFS or OS in recurrent grade II or grade III meningioma (80). Thus, with disappointing results from cytotoxic chemotherapies, many are looking for molecularly-targeted drugs to improve outcomes.

### Molecularly-Targeted Therapies

The *NF2* gene product, Merlin, is known to negatively regulate mTOR signaling (81). In meningioma cell lines and patient samples deficient in Merlin, constitutive activation of mTOR signaling and overexpression of mTORC1 has been observed (81, 82). As such, drugs targeting the mTOR hold promise for treatment of *NF2*-mutated meningiomas. Everolimus, an mTOR inhibitor FDA approved for several other cancers, was trialed in vestibular schwannoma with data showing no radiographic evidence of response. However, a more recent effort, which includes meningioma is still underway (NCT01880749). While a phase II trial of everolimus in combination with bevacizumab showed little improvement in PFS in comparison to treatment with bevacizumab alone (NCT00972335) (83), the combination of everolimus and octreotide decreased tumor growth rate by more than 50% in 29/35 patients (NCT02333565) (84). Improved PFS was also noted, and likely merits further study. The lack of radiographic response or evidence of apoptosis in response to mTOR inhibition does lessen enthusiasm for this approach. However, a second mTOR inhibitor, AZD2014 or vistusertib, is also under evaluation for use in meningioma (NCT03071874 and NCT02831257).

The PI3K and AKT signaling pathways function upstream of mTOR and, as previously mentioned, have been identified as common and potentially targetable mutations. An upcoming trial will examine the effects of the PI3K inhibitor, Alpelisib, in combination with the MEK inhibitor, Trametinib (NCT03631953). Similar to inhibition of mTOR, blockade of PI3K signaling may not induce apoptosis. However, *in vitro* data from primary meningioma cell lines demonstrates caspase-induced cell death via MEK inhibition. As such, this combination therapy may prove effective.

The recent explosion in cancer immunotherapy has, of course, brought to light the potential of PD-1 and PD-L1 targeting antibodies for treatment of meningioma. As with glioma, meningioma often presents with signs of T cell exhaustion and immune evasion, which can lead to decreased levels of PD-1^+^ T cells. However, trials of the inhibitory PD-1 antibody-based therapies, prembrolizumab (NCT03016091, NCT03279692), nivolumab alone (NCT02648997), or in combination with ipilumumab (CTLA4 inhibitor NCT03604978) are ongoing. A recent case report demonstrated remarkable response to nivolumab in a patient with recurrent, treatment-refractory meningioma and homozygous deletion of the DNA mismatch repair gene, MSH2 (85). Dysfunction of mismatch repair mechanisms is associated with increased mutational burden and presence of tumor neoantigens, which may lead to heightened immune response. As such, further investigation of MSH2, anti-PD-1 antibodies, and other immunotherapy-based approaches is warranted.

As an alternative to inhibition of the PD-1 receptor, antibody-based therapies targeting the PD-1 ligand, PD-L1 are also in clinical trials for many types of tumors. Indeed, PD-L1 is upregulated in many meningiomas, especially grade II and III tumors. *In vitro* studies using meningioma and natural killer (NIK) cell lines demonstrated that the anti-PD-L1 antibody, avelumab, can induce antibody-dependent cellular cytotoxicity (86). In an ongoing clinical trial, the efficacy of avelumab as an adjuvant therapy with hypofractionated proton radiation is being tested in recurrent radiation-refractory patients (NCT03267836).

For patients with aggressive rhabdoid meningioma harboring BAP1 mutations, Tazemetostat, which awaits FDA approval for other tumor types, may prove useful. In mesothelioma, inactivation of BAP1 leads to increased levels of the PCR2 complex protein, EZH2 (87), the target of Tazemetostat. Thus, use of this drug in meningioma patients with BAP1 driver mutations may prove clinically beneficial in future trials (88). In addition, downstream dysregulation of the PCR2 complex as a result of SWI/SNF mutations (38), suggest Tazemetostat may also benefit these patients. Aside from these mutations, the hypermethylation of numerous genes in high grade meningiomas may merit trials of EZH2 inhibitors across a wide range of patients.

### Novel Approaches

As an alternative to octreotide and pasireotide therapy, somatostatin receptor analogs can also be radiolabeled and administered in an experimental approach referred to as peptide receptor radionuclide therapy (PRRT). The somatostatin receptor analogs are used to direct radionuclides for uptake by tumor cells, leading to death in a targeted fashion. Preliminary data using copper-64 labeled SARTATE suggests it can be safe and effective in neuroendocrine tumors (89). This radionuclide, and others including 177Lu-DOTA-Tyr3-octreotate (Lutathera), are currently in trials for meningioma (NCT03936426, NCT03971461).

Separate from drug-based management of meningioma, two current trials are examining the utility of tumor-treating fields, such as the NovoTTF-110A, now known as Optune. This approach utilizes scalp-attached patches to send low intensity electrical fields to the brain. In patients with glioblastoma, these fields are known to interfere with tumor cell division and growth, thereby improving quality of life and prolonging survival. Current efforts are testing the efficacy of Optune alone and in combination with bevacizumab therapy (NCT01892397, NCT02847559).

## Discussion

Though commonly thought of as benign tumors, meningiomas present an enduring challenge for physicians. Subjective WHO grading and extensive variability in the propensity of, and time to recurrence, makes the choice of an optimal therapeutic approach difficult. As with most intracranial tumors, maximal safe surgical resection is highly effective, but in inoperable cases, those in which residual tumor remains, and aggressive high-grade cases, adjuvant therapy is required. Unfortunately, few effective systemic therapies are approved for use.

To increase the arsenal of effective therapies, researchers have turned to cytogenetic, next-generation sequencing, and immunohistochemical analyses to identify new molecular drug targets. However, given the generally low mutational burden of meningioma, early efforts to identify prognostic biomarkers yielded few tangible improvements in patient outcomes. More recently, mutations identified in the *TERT* promoter and *BAP1* were linked to poor prognosis, while *FGFR3* mutations were suggestive of improved outcome. Unfortunately, each of these mutations occurs at low frequency and thus the majority of patients are not impacted. Protein level biomarkers regulated by epigenetic changes represent a promising and growing area of interest with respect to meningioma. Indeed, increasing tumor grade is associated with increased expression of VEGF, Ki67, TOP2, PD-1, and PDGFRB (17, 48, 53), while hypermethylation of *RIZ1* and *WNK2* leads to loss of protein expression in high grade meningioma (6, 41). In addition, the methylation status of several T-box and Hox genes may also predict poor outcomes independent of gene expression (42, 44), thus bolstering the support for methylation profiling as a means to predict recurrence in meningioma (44).

The effort to identify new informative mutations and protein biomarkers has driven forward new clinical trials for treatment of meningioma. While cytotoxic chemotherapies have failed to significantly extend progression free and overall survival, some targeted therapies seem promising. Cytostatic mTOR inhibitors show promise in controlling tumor growth, though the lack of cytotoxic effects suggests these may be better suited for use in combination therapies. Though yet to be tested in randomized trials, inhibition of EZH2 has the potential to improve outcomes for several groups of patients. Overall, focused research and implementation of new approaches, including PRRT and TTF devices, will hopefully translate to improved outcomes for meningioma patients in the coming years.

## Author Contributions

MA-R and KF collected, analyzed the clinical data, and wrote part of the manuscript. MA designed the study, collected the data, wrote and edited the manuscript. All authors approved the final manuscript.

### Conflict of Interest

The authors declare that the research was conducted in the absence of any commercial or financial relationships that could be construed as a potential conflict of interest.

## References

[1] Wiemels J Wrensch M Claus EB. Epidemiology and etiology of meningioma. J Neurooncol. (2010) 99:307–14. 10.1007/s11060-010-0386-3 20821343 PMC2945461

[2] Rohringer M Sutherland GR Louw DF Sima AA. Incidence and clinicopathological features of meningioma. J Neurosurg. (1989) 71:665–72. 10.3171/jns.1989.71.5.0665 2809720

[3] Proctor DT Ramachandran S Lama S Sutherland GR. Towards molecular classification of meningioma: evolving treatment and diagnostic paradigms. World Neurosurg. (2018) 119:366–73. 10.1016/j.wneu.2018.08.019 30138732

[4] Louis DN Perry A Reifenberger G von Deimling A Figarella-Branger D Cavenee WK. The 2016 World Health Organization classification of tumors of the central nervous system: a summary. Acta Neuropathol. (2016) 131:803–20. 10.1007/s00401-016-1545-1 27157931

[5] Kalamarides M Stemmer-Rachamimov AO Niwa-Kawakita M Chareyre F Taranchon E Han ZY. Identification of a progenitor cell of origin capable of generating diverse meningioma histological subtypes. Oncogene. (2011) 30:2333–44. 10.1038/onc.2010.609 21242963

[6] Liu ZY Wang JY Liu HH Ma XM Wang CL Zhang XP. Retinoblastoma protein-interacting zinc-finger gene 1 (RIZ1) dysregulation in human malignant meningiomas. Oncogene. (2013) 32:1216–22. 10.1038/onc.2012.155 22614009

[7] Bi WL Zhang M Wu WW Mei Y Dunn IF. Meningioma genomics: diagnostic, prognostic, and therapeutic applications. Front Surg. (2016) 3:40. 10.3389/fsurg.2016.00040 27458586 PMC4933705

[8] Liu Z Wang C Wang H Wang Y Li JY Liu Y. Clinical characteristics and treatment of angiomatous meningiomas: a report of 27 cases. Int J Clin Exp Pathol. (2013) 6:695–702. 23573316 PMC3606859

[9] Domingues PH Sousa P Otero A Goncalves JM Ruiz L de Oliveira C. Proposal for a new risk stratification classification for meningioma based on patient age, WHO tumor grade, size, localization, and karyotype. Neuro Oncol. (2014) 16:735–47. 10.1093/neuonc/not325 24536048 PMC3984558

[10] Lerner C Ketter R Linsler S Henn W Oertel J Urbschat S. Establishment of a molecular cytogenetic analysis for native tumor tissue of meningiomas-suitable for clinical application. Mol Cytogenet. (2014) 7:12. 10.1186/1755-8166-7-12 24499596 PMC3937053

[11] Bi WL Greenwald NF Abedalthagafi M Wala J Gibson WJ Agarwalla PK Genomic landscape of high-grade meningiomas. NPJ Genom Med. (2017) 2:15 10.1038/s41525-017-0014-7 28713588 PMC5506858

[12] Brastianos PK Horowitz PM Santagata S Jones RT McKenna A Getz G. Genomic sequencing of meningiomas identifies oncogenic SMO and AKT1 mutations. Nat Genet. (2013) 45:285–9. 10.1038/ng.2526 23334667 PMC3739288

[13] Lee Y Liu J Patel S Cloughesy T Lai A Farooqi H. Genomic landscape of meningiomas. Brain Pathol. (2010) 20:751–62. 10.1111/j.1750-3639.2009.00356.x 20015288 PMC3167483

[14] Ruttledge MH Sarrazin J Rangaratnam S Phelan CM Twist E Merel P. Evidence for the complete inactivation of the NF2 gene in the majority of sporadic meningiomas. Nat Genet. (1994) 6:180–4. 10.1038/ng0294-180 8162072

[15] Sulman EP White PS Brodeur GM. Genomic annotation of the meningioma tumor suppressor locus on chromosome 1p34. Oncogene. (2004) 23:1014–20. 10.1038/sj.onc.1206623 14749765

[16] Goutagny S Yang HW Zucman-Rossi J Chan J Dreyfuss JM Park PJ. Genomic profiling reveals alternative genetic pathways of meningioma malignant progression dependent on the underlying NF2 status. Clin Cancer Res. (2010) 16:4155–64. 10.1158/1078-0432.CCR-10-0891 20682713

[17] Everson RG Hashimoto Y Freeman JL Hodges TR Huse J Zhou S. Multiplatform profiling of meningioma provides molecular insight and prioritization of drug targets for rational clinical trial design. J Neurooncol. (2018) 139:469–78. 10.1007/s11060-018-2891-8 29846894

[18] Abedalthagafi MS Merrill PH Bi WL Jones RT Listewnik ML Ramkissoon SH. Angiomatous meningiomas have a distinct genetic profile with multiple chromosomal polysomies including polysomy of chromosome 5. Oncotarget. (2014) 5:10596–606. 10.18632/oncotarget.2517 25347344 PMC4279396

[19] Clark VE Erson-Omay EZ Serin A Yin J Cotney J Ozduman K. Genomic analysis of non-NF2 meningiomas reveals mutations in TRAF7, KLF4, AKT1, and SMO. Science. (2013) 339:1077–80. 10.1126/science.1233009 23348505 PMC4808587

[20] Sahm F Schrimpf D Olar A Koelsche C Reuss D Bissel J. TERT promoter mutations and risk of recurrence in meningioma. J Natl Cancer Inst. (2016) 108:djv377. 10.1093/jnci/djv377 26668184 PMC4849806

[21] Spiegl-Kreinecker S Lotsch D Neumayer K Kastler L Gojo J Pirker C. TERT promoter mutations are associated with poor prognosis and cell immortalization in meningioma. Neuro Oncol. (2018) 20:1584–93. 10.1093/neuonc/noy104 30010853 PMC6231195

[22] Biczok A Kraus T Suchorska B Terpolilli NA Thorsteinsdottir J Giese A. TERT promoter mutation is associated with worse prognosis in WHO grade II and III meningiomas. J Neurooncol. (2018) 139:671–8. 10.1007/s11060-018-2912-7 29808339

[23] AlSahlawi A Aljelaify R Magrashi A AlSaeed M Almutairi A Alqubaishi F. New insights into the genomic landscape of meningiomas identified FGFR3 in a subset of patients with favorable prognoses. Oncotarget. (2019) 10:5549–59. 10.18632/oncotarget.27178 31565188 PMC6756861

[24] Yuzawa S Nishihara H Tanaka S. Genetic landscape of meningioma. Brain Tumor Pathol. (2016) 33:237–47. 10.1007/s10014-016-0271-7 27624470

[25] Zotti T Scudiero I Vito P Stilo R. The emerging role of TRAF7 in tumor development. J Cell Physiol. (2017) 232:1233–8. 10.1002/jcp.25676 27808423 PMC5347962

[26] Abedalthagafi M Bi WL Aizer AA Merrill PH Brewster R Agarwalla PK. Oncogenic PI3K mutations are as common as AKT1 and SMO mutations in meningioma. Neuro Oncol. (2016) 18:649–55. 10.1093/neuonc/nov316 26826201 PMC4827048

[27] Bleeker FE Felicioni L Buttitta F Lamba S Cardone L Rodolfo M. AKT1(E17K) in human solid tumours. Oncogene. (2008) 27:5648–50. 10.1038/onc.2008.170 18504432

[28] Boetto J Bielle F Sanson M Peyre M Kalamarides M. SMO mutation status defines a distinct and frequent molecular subgroup in olfactory groove meningiomas. Neuro Oncol. (2017) 19:345–51. 10.1093/neuonc/now276 28082415 PMC5464306

[29] Mawrin C Sasse T Kirches E Kropf S Schneider T Grimm C. Different activation of mitogen-activated protein kinase and Akt signaling is associated with aggressive phenotype of human meningiomas. Clin Cancer Res. (2005) 11:4074–82. 10.1158/1078-0432.CCR-04-2550 15930342

[30] Tang H Zhu H Wang X Hua L Li J Xie Q. KLF4 is a tumor suppressor in anaplastic meningioma stem-like cells and human meningiomas. J Mol Cell Biol. (2017) 9:315–24. 10.1093/jmcb/mjx023 28651379

[31] Reuss DE Piro RM Jones DT Simon M Ketter R Kool M. Secretory meningiomas are defined by combined KLF4 K409Q and TRAF7 mutations. Acta Neuropathol. (2013) 125:351–8. 10.1007/s00401-013-1093-x 23404370

[32] Schuetz A Nana D Rose C Zocher G Milanovic M Koenigsmann J. The structure of the Klf4 DNA-binding domain links to self-renewal and macrophage differentiation. Cell Mol Life Sci. (2011) 68:3121–31. 10.1007/s00018-010-0618-x 21290164 PMC11114807

[33] Clark VE Harmanci AS Bai H Youngblood MW Lee TI Baranoski JF. Recurrent somatic mutations in POLR2A define a distinct subset of meningiomas. Nat Genet. (2016) 48:1253–9. 10.1038/ng.3651 27548314 PMC5114141

[34] Gerkes EH Fock JM den Dunnen WF van Belzen MJ van der Lans CA Hoving EW. A heritable form of SMARCE1-related meningiomas with important implications for follow-up and family screening. Neurogenetics. (2016) 17:83–9. 10.1007/s10048-015-0472-y 26803492 PMC4794526

[35] Smith MJ Wallace AJ Bennett C Hasselblatt M Elert-Dobkowska E Evans LT. Germline SMARCE1 mutations predispose to both spinal and cranial clear cell meningiomas. J Pathol. (2014) 234:436–40. 10.1002/path.4427 25143307

[36] van den Munckhof P Christiaans I Kenter SB Baas F Hulsebos TJ. Germline SMARCB1 mutation predisposes to multiple meningiomas and schwannomas with preferential location of cranial meningiomas at the falx cerebri. Neurogenetics. (2012) 13:1–7. 10.1007/s10048-011-0300-y 22038540

[37] Shankar GM Abedalthagafi M Vaubel RA Merrill PH Nayyar N Gill CM. Germline and somatic BAP1 mutations in high-grade rhabdoid meningiomas. Neuro Oncol. (2017) 19:535–45. 10.1093/neuonc/now235 28170043 PMC5464371

[38] Collord G Tarpey P Kurbatova N Martincorena I Moran S Castro M. An integrated genomic analysis of anaplastic meningioma identifies prognostic molecular signatures. Sci Rep. (2018) 8:13537. 10.1038/s41598-018-31659-0 30202034 PMC6131140

[39] Xue Y Chen R Du W Yang F Wei X. RIZ1 and histone methylation status in pituitary adenomas. Tumour Biol. (2017) 39:1010428317711794. 10.1177/1010428317711794 28718376

[40] Shaikh N Dixit K Raizer J. Recent advances in managing/understanding meningioma. F1000Res. (2018) 7:490. 10.12688/f1000research.13674.1 29770198 PMC5931261

[41] Jun P Hong C Lal A Wong JM McDermott MW Bollen AW. Epigenetic silencing of the kinase tumor suppressor WNK2 is tumor-type and tumor-grade specific. Neuro Oncol. (2009) 11:414–22. 10.1215/15228517-2008-096 19001526 PMC2743221

[42] Bi WL Abedalthagafi M Horowitz P Agarwalla PK Mei Y Aizer AA. Genomic landscape of intracranial meningiomas. J Neurosurg. (2016) 125:525–35. 10.3171/2015.6.JNS15591 26771848

[43] Sahm F Schrimpf D Stichel D Jones DTW Hielscher T Schefzyk S. DNA methylation-based classification and grading system for meningioma: a multicentre, retrospective analysis. Lancet Oncol. (2017) 18:682–94. 10.1016/S1470-2045(17)30155-9 28314689

[44] Nassiri F Mamatjan Y Suppiah S Badhiwala JH Mansouri S Karimi S DNA methylation profiling to predict recurrence risk in meningioma: development and validation of a nomogram to optimize clinical management. Neuro Oncol. (2019) 21:901–10. 10.1093/neuonc/noz061 31158293 PMC6620635

[45] Boulagnon-Rombi C Fleury C Fichel C Lefour S Bressenot AM Gauchotte G. Immunohistochemical approach to the differential diagnosis of meningiomas and their mimics. J Neuropathol Exp Neurol. (2017) 76:289–98. 10.1093/jnen/nlx008 28340171

[46] Ng J Celebre A Munoz DG Keith JL Karamchandani JR. Sox10 is superior to S100 in the diagnosis of meningioma. Appl Immunohistochem Mol Morphol. (2015) 23:215–9. 10.1097/PAI.0000000000000072 25265429

[47] Berghoff AS Kresl P Bienkowski M Koelsche C Rajky U Hainfellner JA. Validation of nuclear STAT6 immunostaining as a diagnostic marker of meningeal solitary fibrous tumor (SFT)/hemangiopericytoma. Clin Neuropathol. (2017) 36:56–9. 10.5414/NP300993 28128724

[48] Lee SH Lee YS Hong YG Kang CS. Significance of COX-2 and VEGF expression in histopathologic grading and invasiveness of meningiomas. APMIS. (2014) 122:16–24. 10.1111/apm.12079 23756256

[49] Backer-Grondahl T Moen BH Arnli MB Torseth K Torp SH. Immunohistochemical characterization of brain-invasive meningiomas. Int J Clin Exp Pathol. (2014) 7:7206–19. 25400818 PMC4230100

[50] Reszec J Hermanowicz A Rutkowski R Turek G Mariak Z Chyczewski L. Expression of MMP-9 and VEGF in meningiomas and their correlation with peritumoral brain edema. Biomed Res Int. (2015) 2015:646853. 10.1155/2015/646853 25821815 PMC4363610

[51] Wernicke AG Dicker AP Whiton M Ivanidze J Hyslop T Hammond EH. Assessment of epidermal growth factor receptor (EGFR) expression in human meningioma. Radiat Oncol. (2010) 5:46. 10.1186/1748-717X-5-46 20509969 PMC2890616

[52] Arnli MB Backer-Grondahl T Ytterhus B Granli US Lydersen S Gulati S. Expression and clinical value of EGFR in human meningiomas. PeerJ. (2017) 5:e3140. 10.7717/peerj.3140 28367377 PMC5374971

[53] Yang SY Xu GM. Expression of PDGF and its receptor as well as their relationship to proliferating activity and apoptosis of meningiomas in human meningiomas. J Clin Neurosci. (2001) 8:49–53. 10.1054/jocn.2001.0877 11386826

[54] Fang L Lowther DE Meizlish ML Anderson RC Bruce JN Devine L. The immune cell infiltrate populating meningiomas is composed of mature, antigen-experienced T and B cells. Neuro Oncol. (2013) 15:1479–90. 10.1093/neuonc/not110 23978377 PMC3813416

[55] Du Z Abedalthagafi M Aizer AA McHenry AR Sun HH Bray MA. Increased expression of the immune modulatory molecule PD-L1 (CD274) in anaplastic meningioma. Oncotarget. (2015) 6:4704–16. 10.18632/oncotarget.3082 25609200 PMC4467109

[56] Zou W. Regulatory T cells, tumour immunity and immunotherapy. Nat Rev Immunol. (2006) 6:295–307. 10.1038/nri1806 16557261

[57] Han SJ Reis G Kohanbash G Shrivastav S Magill ST Molinaro AM. Expression and prognostic impact of immune modulatory molecule PD-L1 in meningioma. J Neurooncol. (2016) 130:543–52. 10.1007/s11060-016-2256-0 27624915 PMC5560602

[58] Johnson MD. PD-L1 expression in meningiomas. J Clin Neurosci. (2018) 57:149–51. 10.1016/j.jocn.2018.08.023 30153998

[59] Pinton L Solito S Masetto E Vettore M Cane S Puppa AD. Immunosuppressive activity of tumor-infiltrating myeloid cells in patients with meningioma. Oncoimmunology. (2018) 7:e1440931. 10.1080/2162402X.2018.1440931 29900047 PMC5993508

[60] Gupta S Bi WL Dunn IF. Medical management of meningioma in the era of precision medicine. Neurosurg Focus. (2018) 44:E3. 10.3171/2018.1.FOCUS17754 29606052

[61] Schrell UM Rittig MG Anders M Koch UH Marschalek R Kiesewetter F. Hydroxyurea for treatment of unresectable and recurrent meningiomas. II. Decrease in the size of meningiomas in patients treated with hydroxyurea. J Neurosurg. (1997) 86:840–4. 10.3171/jns.1997.86.5.0840 9126900

[62] Swinnen LJ Rankin C Rushing EJ Laura HF Damek DM Barger GR Phase II study of hydroxyurea for unresectable meningioma (Southwest Oncology Group S9811). J Clin Oncol. (2009) 27:2063.

[63] Kim J Kim KH Kim YZ. The clinical outcome of hydroxyurea chemotherapy after incomplete resection of atypical meningiomas. Brain Tumor Res Treat. (2017) 5:77–86. 10.14791/btrt.2017.5.2.77 29188208 PMC5700031

[64] Chamberlain MC Tsao-Wei DD Groshen S. Temozolomide for treatment-resistant recurrent meningioma. Neurology. (2004) 62:1210–2. 10.1212/01.WNL.0000118300.82017.F4 15079029

[65] Grunberg SM Weiss MH Spitz IM Ahmadi J Sadun A Russell CA. Treatment of unresectable meningiomas with the antiprogesterone agent mifepristone. J Neurosurg. (1991) 74:861–6. 10.3171/jns.1991.74.6.0861 2033444

[66] Ji Y Rankin C Grunberg S Sherrod AE Ahmadi J Townsend JJ. Double-blind phase III randomized trial of the antiprogestin agent mifepristone in the treatment of unresectable meningioma: SWOG S9005. J Clin Oncol. (2015) 33:4093–8. 10.1200/jco.2015.33.15_suppl.e17084 26527781 PMC4669593

[67] Grunberg SM Weiss MH Russell CA Spitz IM Ahmadi J Sadun A. Long-term administration of mifepristone (RU486): clinical tolerance during extended treatment of meningioma. Cancer Invest. (2006) 24:727–33. 10.1080/07357900601062339 17162554

[68] Lamberts SW Tanghe HL Avezaat CJ Braakman R Wijngaarde R Koper JW. Mifepristone (RU 486) treatment of meningiomas. J Neurol Neurosurg Psychiatry. (1992) 55:486–90. 10.1136/jnnp.55.6.486 1619417 PMC1014906

[69] Norden AD Raizer JJ Abrey LE Lamborn KR Lassman AB Chang SM. Phase II trials of erlotinib or gefitinib in patients with recurrent meningioma. J Neurooncol. (2010) 96:211–7. 10.1007/s11060-009-9948-7 19562255 PMC3786190

[70] Wen PY Yung WK Lamborn KR Norden AD Cloughesy TF Abrey LE. Phase II study of imatinib mesylate for recurrent meningiomas (North American Brain Tumor Consortium study 01-08). Neuro Oncol. (2009) 11:853–60. 10.1215/15228517-2009-010 19293394 PMC2802405

[71] Kaley TJ Wen P Schiff D Ligon K Haidar S Karimi S. Phase II trial of sunitinib for recurrent and progressive atypical and anaplastic meningioma. Neuro Oncol. (2015) 17:116–21. 10.1093/neuonc/nou148 25100872 PMC4483051

[72] Nunes FP Merker VL Jennings D Caruso PA di Tomaso E Muzikansky A. Bevacizumab treatment for meningiomas in NF2: a retrospective analysis of 15 patients. PLoS ONE. (2013) 8:e59941. 10.1371/journal.pone.0059941 23555840 PMC3605344

[73] Grimm SA Kumthekar P Chamberlain MC Schiff D Wen PY Iwamoto FM Phase II trial of bevacizumab in patients with surgery and radiation refractory progressive meningioma. J Clin Oncol. (2015) 33:2055 10.1200/jco.2015.33.15_suppl.2055

[74] Schulz C Mathieu R Kunz U Mauer UM. Treatment of unresectable skull base meningiomas with somatostatin analogs. Neurosurg Focus. (2011) 30:E11. 10.3171/2011.1.FOCUS111 21529167

[75] Johnson DR Kimmel DW Burch PA Cascino TL Giannini C Wu W. Phase II study of subcutaneous octreotide in adults with recurrent or progressive meningioma and meningeal hemangiopericytoma. Neuro Oncol. (2011) 13:530–5. 10.1093/neuonc/nor044 21558077 PMC3093340

[76] Simo M Argyriou AA Macia M Plans G Majos C Vidal N. Recurrent high-grade meningioma: a phase II trial with somatostatin analogue therapy. Cancer Chemother Pharmacol. (2014) 73:919–23. 10.1007/s00280-014-2422-z 24619496

[77] Norden AD Ligon KL Hammond SN Muzikansky A Reardon DA Kaley TJ Phase II study of monthly pasireotide LAR (SOM230C) for recurrent or progressive meningioma. Neurology. (2015) 84:280–6. 10.1212/WNL.0000000000001153 25527270 PMC4335993

[78] Graillon T Romano D Defilles C Saveanu A Mohamed A Figarella-Branger D. Octreotide therapy in meningiomas: *in vitro* study, clinical correlation, and literature review. J Neurosurg. (2017) 127:660–9. 10.3171/2016.8.JNS16995 27982767

[79] Preusser M Spiegl-Kreinecker S Lotsch D Wohrer A Schmook M Dieckmann K. Trabectedin has promising antineoplastic activity in high-grade meningioma. Cancer. (2012) 118:5038–49. 10.1002/cncr.27460 22392434

[80] Preusser M Silvani A Le Rhun E Soffietti R Lombardi G Sepúlveda JM Trabectedin for recurrent WHO grade II or III meningioma: a randomized phase II study of the EORTC brain tumor group (EORTC-1320-BTG). J Clin Oncol. (2019) 37:2007 10.1200/JCO.2019.37.15_suppl.2007 PMC9071312 34672349

[81] James MF Han S Polizzano C Plotkin SR Manning BD Stemmer-Rachamimov AO. NF2/merlin is a novel negative regulator of mTOR complex 1, and activation of mTORC1 is associated with meningioma and schwannoma growth. Mol Cell Biol. (2009) 29:4250–61. 10.1128/MCB.01581-08 19451225 PMC2715803

[82] James MF Stivison E Beauchamp R Han S Li H Wallace MR. Regulation of mTOR complex 2 signaling in neurofibromatosis 2-deficient target cell types. Mol Cancer Res. (2012) 10:649–59. 10.1158/1541-7786.MCR-11-0425-T 22426462

[83] Shih KC Chowdhary S Rosenblatt P Weir AB Shepard GC Williams JT. A phase II trial of bevacizumab and everolimus as treatment for patients with refractory, progressive intracranial meningioma. J Neurooncol. (2016) 129:281–8. 10.1007/s11060-016-2172-3 27311730

[84] Graillon T Sanson M Peyre M Peyrière H Autran D Kalamarides M A phase II of everolimus and octreotide for patients with refractory and documented progressive meningioma (CEVOREM). J Clin Oncol. (2017) 35:2011 10.1200/JCO.2017.35.15_suppl.2011

[85] Dunn IF Du Z Touat M Sisti MB Wen PY Umeton R. Mismatch repair deficiency in high-grade meningioma: a rare but recurrent event associated with dramatic immune activation and clinical response to PD-1 blockade. JCO Precis Oncol. (2018). [Epub ahead of print]. 10.1200/PO.18.00190. 30801050 PMC6383717

[86] Giles AJ Hao S Padget MR Song H Zhang W Lynes J Efficient ADCC- killing of meningioma by avelumab and a high-affinity natural killer cell line, haNK. JCI Insight. (2019) 130688 10.1172/jci.insight.130688 PMC6824312 31536478

[87] LaFave LM Beguelin W Koche R Teater M Spitzer B Chramiec A. Loss of BAP1 function leads to EZH2-dependent transformation. Nat Med. (2015) 21:1344–9. 10.1038/nm.3947 26437366 PMC4636469

[88] Shankar GM Santagata S. BAP1 mutations in high-grade meningioma: implications for patient care. Neuro Oncol. (2017) 19:1447–56. 10.1093/neuonc/nox094 28482042 PMC5737364

[89] Hicks RJ Jackson P Kong G Ware RE Hofman MS Pattison DA First-in-human trial of ^64^Cu-SARTATE PET imaging of patients with neuroendocrine tumors demonstrates high tumor uptake and retention, potentially allowing prospective dosimetry for peptide receptor radionuclide therapy. J Nucl Med. (2018) 60:777–85. 10.2967/jnumed.118.217745 30442752 PMC6581229

